# Effects of *peri*-arrest hypertonic/hyperoncotic fluid treatment on clinical outcomes in non-trauma-related resuscitation – A scoping review

**DOI:** 10.1016/j.resplu.2025.101171

**Published:** 2025-11-20

**Authors:** Samira Akbas, Clara Castellucci, Christopher Haresleb, Alexander Kaserer, Donat R. Spahn, Jan Breckwoldt

**Affiliations:** a Institute of Anaesthesiology and Perioperative Medicine, University Hospital Zurich, University of Zurich, Raemistrasse 100, 8091 Zurich, Switzerland; b Division of Cardiothoracic and Vascular Anesthesia & Critical Care Medicine, Department of Anesthesia, General Intensive Care and Pain Management, Medical University of Vienna, Waehringer Guertel 18-20, 1090 Vienna, Austria; c Department of Anaesthesiology, Kantonsspital Baselland, Rheinstrasse 26, 4410 Liestal, Switzerland; d University of Zurich, Zurich, Switzerland

**Keywords:** Scoping review, Hypertonic fluids, Hyperoncotic fluids, Out-of-hospital cardiac arrest, Cardiopulmonary resuscitation

## Abstract

**Aim:**

This scoping review aimed to summarize existing evidence on the administration of hypertonic and hyperoncotic (HTF/HOF) fluids during cardiopulmonary resuscitation (CPR) in both human and animal studies. Further, we sought to provide guidance for future research and potential clinical implementation strategies.

**Methods:**

We searched *Medline, EMBASE, Cochrane, Web of Science, Scopus and**Clinicaltrials.gov* from inception to June 11th, 2025, for comparative studies using HTF/HOF in non-trauma-related *peri*-arrest settings in animal models and humans. Studies were selected using a PICOST format. Studies were assessed on their risk of bias. Results were synthesized narratively and presented using descriptive statistics. No meta-analysis was performed.

**Results:**

We included 21 animal and four human studies. Animal studies showed high heterogeneity in species, cardiac arrest models, timing and composition of HTF/HOF. Most of them found improved neurological outcomes compared to normal saline. In the human studies (overall sample size 932), patients were either treated with a combination of hypertonic saline and hydroxyethyl starch (HHS) or with hypertonic saline alone (HS). Time points of fluid administration and assessed outcome parameters differed substantially. Overall, human studies indicated neutral to favorable effects, supporting the potential value of HTF/HOF treatment.

**Conclusions:**

Studies analyzing the effects of HTF/HOF in *peri*-arrest settings in animals and in humans suggest potential benefit on survival and neurological outcomes. Findings are limited by substantial heterogeneity in study designs, timing and outcome measures. It therefore appears necessary to conduct more rigorous clinical trials with standardized endpoints to further explore the potential of *peri*-arrest HTF/HOF treatment.

## Introduction

Cardiac arrest is a major cause of death, and its rate of survival with favorable cerebral outcome is low.^1^, ^2^ For improving outcomes, coronary and cerebral blood flow must be maintained during cardiopulmonary resuscitation (CPR).^3^ To underscore this principle, the concept of cardio-cerebral resuscitation was introduced pointing at cerebral perfusion as the main target of CPR.^4^, ^5^ In this context, reperfusion injury has been identified as a significant threat to cerebral outcome. With cerebral perfusion being dependent on intravascular volume, vascular fluid administration was proposed as a therapeutic concept, peri- and post-cardiac arrest. However, volume expansion might also have negative effects on cardiac function, as studies have shown in post-arrest cooling with large volumes of cold fluid.^6^, ^7^ In contrast, hypertonic and hyperoncotic fluids are of small volume, and act by recruiting fluid from the interstitial space into the blood vessels, thereby inducing transient plasma volume expansion.^8^, ^9^ Hypertonic fluids, such as hypertonic saline, exert osmotic effects by drawing fluid from surrounding tissues into blood vessels thereby increasing circulating volume and perfusion pressure. Some of these promising results have been transferred to human studies with five trials investigating hypertonic saline or hypertonic saline/dextrane during resuscitation.^10^, ^11^ Taken together, targeted administration of hypertonic or hyperoncotic fluids may support cerebral perfusion by transiently increasing the stressed intravascular volume.

A second, important problem induced by circulatory arrest is cerebral hypoxia, causing endothelial cell swelling and perivascular oedema,^12^, ^13^ with the effect of impeding reperfusion and rapid re-oxygenation of hypoxia-sensitive neurons. CPR generates only a low cerebral blood flow, which is insufficient to sustain cellular integrity.^14^ Once return of spontaneous circulation (ROSC) is achieved, cerebral blood flow is restored with reperfusion of ischemic areas. Paradoxically, this process catalyzes multiple mechanisms causing secondary brain injury promoting further neuronal damage, known as reperfusion injury.^15^

Administration of hypertonic-hyperoncotic saline has been shown in animal studies to improve microcirculatory reperfusion in the heart and brain, leading to better short-term survival and neurological recovery.^16^ Some of these promising results have been transferred to human studies and administration of hypertonic-hyperoncotic solutions has been shown safe. A multicentre randomized trial is ongoing to evaluate the administration of hypertonic sodium lactate immediately after ROSC in humans (NCT05004610), reflecting growing clinical interest in hypertonic fluids. However, sample sizes of the past studies were limited^17^, ^18^ and a consensus on specific fluid therapy to support cerebral blood flow and prevent further cerebral damage has not yet been established. But hypertonic-hyperoncotic solutions are easily accessible, easily applicable and an economic choice. It therefore appears justified to summarize the current evidence to provide a more solid basis for undertaking further research in this clinical field.

This scoping review maps the existing evidence on the administration of hypertonic and hyperoncotic fluids during or immediately after non-trauma related cardiac arrest. The aim is to identify knowledge gaps to inform further research.

## Methods

### Study design

This review was prospectively submitted to the International Prospective Register of Systematic Reviews (PROSPERO) and was not assigned a registration number on October 29th, 2021, considering this review fell under the category of a scoping review. We conducted the review according to the Cochrane methodology and reported findings according to the *Preferred Reporting Items for Systematic reviews and Meta-Analyses extension for Scoping Reviews* (PRISMA-ScR).^19^

### Objectives

The aim of this scoping review was to assess the following outcomes: ROSC; admission to hospital; survival to discharge and survival with favorable outcome at three months and one year as well as neurologic sequelae after use of a defined fluid therapy within context of non-trauma-related resuscitation. Favorable outcome was defined as Cerebral Performance Category (CPC) class 1–2, or equivalent.

### Search strategy

As of June 24th, 2025 there were no pre-existing PICO(ST)s related to the scope of our work on the International Liaison Committee of Resuscitation (ILCOR) Task Force for Advanced Life Support (ALS) website.^20^

Using a pre-determined search strategy developed by an information specialist (available in Supplemental File 1), the databases *Ovid Medline*, *EMBASE*, *Cochrane Library*, *Web of Science*, *Scopus* and *Clinicaltrials.gov* were scanned for eligible articles from beginning of the databases’ existence until a final search update on June 11th, 2025. The initial search was conducted on August 18th, 2021, with follow-up searches on February 21st, 2023, and June 11th, 2025.

### Study selection

We designed the research question based on the Population Intervention Comparator Study design and Timeline (PICOST) concept by the following criteria:

**Population:** Patients or animals receiving non-trauma related CPR in a pre- or in-hospital, or a laboratory setting.

**Intervention:** Any specific/defined fluid therapy using hypertonic or hyperoncotic fluids (HTF/HOFs).

**Comparison:** No specific/defined fluid therapy using HTF/HOFs, or standard fluid therapy.

**Outcomes:** ROSC, admission to hospital, survival to discharge, (neurologic) outcome, survival with favorable outcome at three months and one year.

**Study design:** Randomized controlled trials (RCT) and non-randomized studies (non-randomized controlled trials, controlled before-and-after studies, interrupted time series, matched-pair analyses, cohort studies, published conference abstracts not older than 2 years, and case series where *n* ≥ 5). We included animal studies according to the same criteria. Trial protocols, commentaries, editorials, reviews, meta-analyses and questionnaires were excluded.

**Time frame:** All publications up to June 11, 2025, with an abstract in English.

### Data extraction

All studies retrieved through the database search were imported to Rayyan (rayyan.ai)^21^ and screened to match the previously established PICOST criteria. At least two reviewers (*CC*,*SA, CH, AK* and *JB)* screened each reference. In case of dis-concordance, *CC* and *JB* or *SA* and *JB* established consensus by discussion.

We analyzed and assessed eligible studies on their level of evidence using the Grading of Recommendations Assessment, Development and Evaluation (GRADE)^22^ approach by two independent reviewers. According to the study design, we used the Risk of Bias 2 (RoB 2) tool for randomized controlled trials,^23^ Risk Of Bias In Non-randomized Studies – of Interventions (ROBINS-I) for non-randomized studies^24^ and Systematic Review Center for Laboratory Animal Experimentation (SYRCLE) risk of bias tool for animal studies.^25^

**Ethics:** not applicable.

## Results

**Fig. 1:**
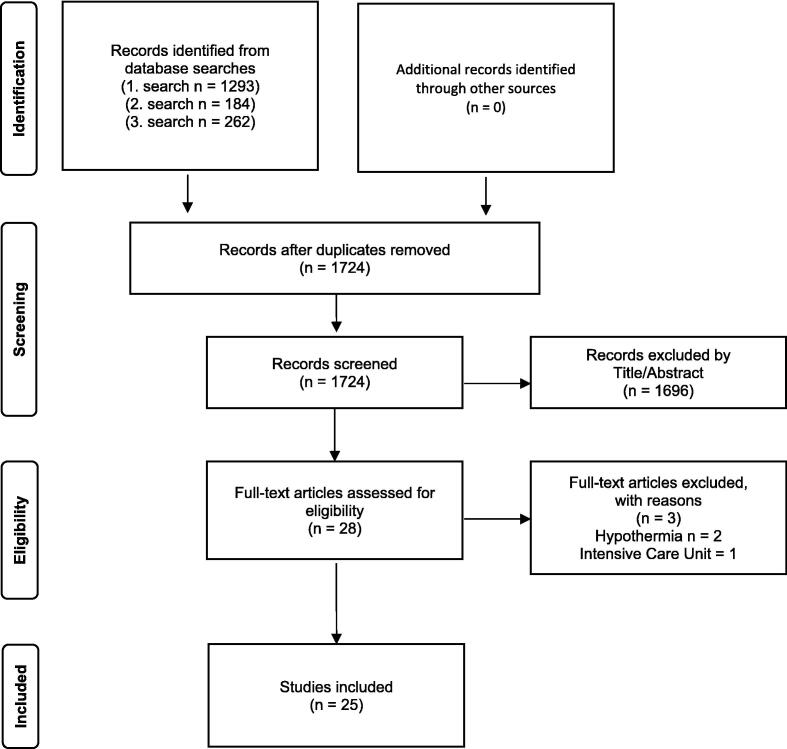
**PRISMA flow diagram for our scoping review**.

The search identified 1′739 abstracts and titles for screening, of which 25 articles fulfilled the predefined PICOST criteria. The PRISMA flow diagram (Fig. 1) shows a detailed description of the selection process. Studies were excluded if they involved trauma-related cardiac arrest, non-comparative designs, pediatric populations or interventions unrelated to hypertonic or hyperoncotic fluids.

### Animal studies

#### Characteristics of studies

Twenty-one studies investigated animal models.^16^, ^26^, ^27^, ^28^, ^29^, ^30^, ^31^, ^32^, ^33^, ^34^, ^35^, ^36^, ^37^, ^38^, ^39^, ^40^, ^41^, ^42^, ^43^, ^44^, ^45^ These studies were performed in swine,^26^, ^27^, ^28^, ^29^, ^30^, ^31^, ^32^, ^33^, ^34^, ^35^, ^36^, ^37^, ^38^, ^45^ rats,^16^, ^39^, ^40^, ^41^ cats^42^, ^43^ and mice.^44^ Cardiac arrest was induced by ventricular fibrillation or asphyxia and CPR was delivered manually or with mechanical devices. Tested fluids included hypertonic saline (HS), hypertonic saline combined with hydroxyethyl starch (HHS), and hydroxyethyl starch (HES),^16^, ^26^, ^27^, ^32^, ^34^, ^35^, ^41^, ^42^, ^43^, ^44^ hypertonic saline with dextrane (HSD),^29^, ^30^, ^31^, ^36^, ^37^, ^38^ two investigated bicarbonate formulations,^33^, ^39^ one hydrogen rich saline^40^ and two did not specify the exact solution.^28^, ^45^ Administration strategies varied: one study infused fluid before cardiac arrest,^16^ one before CPR,^40^ twelve with onset of or during CPR^26^, ^27^, ^28^, ^30^, ^31^, ^33^, ^35^, ^36^, ^37^, ^39^, ^42^, ^45^ and six upon or after ROSC.^29^, ^32^, ^34^, ^38^, ^41^, ^44^ Volumes typically ranged from 2 to 6 ml/kg, with most protocols avoiding large-volume isotonic fluids due to concerns about haemodilution and reduced coronary perfusion.^26^, ^27^, ^30^ Observation periods differed considerably, even within studies with the same animal type, ranging from 20 min^28^ to seven days,^29^, ^40^, ^41^ most commonly observing for several hours.^26^, ^30^, ^31^, ^32^, ^34^, ^35^, ^37^, ^38^, ^39^, ^43^, ^45^ Endpoints also varied: Many trials focused on ROSC and short-term survival,^26^, ^27^, ^29^, ^30^, ^31^, ^39^, ^40^, ^42^ while nine assessed neurological parameters as primary outcomes.^16^, ^28^, ^35^, ^36^, ^37^, ^41^, ^43^, ^44^, ^45^ We summarized study details for animal studies in Table 2.

#### Haemodynamic and perfusion effects in animal models

Several studies demonstrated that early HTF/HOF administration improved mean arterial pressure, cerebral perfusion pressure and global myocardial blood flow during CPR.^26^, ^32^, ^33^, ^45^ Importantly, one landmark study using a feline model showed a significant reduction of cerebral ‘no-reflow’ areas after CPR (from 28 % to 15 % of forebrain area), indicating more homogenous microvascular reperfusion with HTF/HOF therapy.^42^ Others reported improved lactate clearance, less severe acidosis, and earlier ROSC.^27^, ^35^ In addition, several studies suggested organ-protective effects. Treatment with hypertonic solutions reduced myocardial injury markers such as troponin I and decreased indicators of cerebral damage.^32^, ^45^

#### Neuroprotection and cerebral oedema

As a consistent finding across multiple studies, cerebral oedema was attenuated after HTF/HOF treatment. In a mouse model, hypertonic saline reduced brain water content and blood–brain barrier disruption in wild-type but not in α-syntrophin knockout mice, implicating aquaporin-4–mediated water flux an the therapeutic mechanism.^44^ Imaging-based assessments (e.g., MRI, DWI, MRS) further supported that HTF/HOF administration improved ion and water homeostasis, although delayed hypoperfusion was not completely mitigated.^43^ The study by Annoni et al. reported downregulation of neuronal apoptosis, particularly in the hippocampus and cortex, indicating neuroprotective effects.^45^

#### Comparability of animal studies

Apart from the different species, studies varied widely in conceptual design (e.g. asphyctic vs. circulatory arrest), duration of arrest (5–20 min), timing and dosage of fluid administration, and in whether additional measures such as vasopressors, thrombolytics, or targeted temperature management were applied. The administered doses ranged between 2 and 6 ml/kg, corresponding to osmolarities from approximately 1000 to 2400 mOsm/l, depending on the composition. Few studies included long-term survival or functional neurologic evaluation beyond 24–72 h. Finally, two studies did not specify the composition of the hypertonic infusion solution. These methodological differences preclude meaningful data pooling or quantitative comparison of effects. As a result, interpretation must remain descriptive.

#### Risk of bias assessment of animal studies

Animal studies, assessed using the SYCRLE approach, presented mixed risks of bias, mainly in the subgroups of blinding examiners and sample size calculations. The detailed risk of bias assessment is displayed in Supplementary Table 2.

### Human studies

#### Study characteristics

Three of the studies in humans were prospective RCTs^46^, ^47^, ^48^ and one was a retrospective case-control study.^49^ The fluids administered in these studies were roughly comparable. 7.2 % HS combined with 6 % HES 200000/0.5 (HHS) was applied in three studies^46^, ^47^, ^49^ at 2 ml/kg with sample sizes ranging from 66 to 481 patients. One trial analyzed the effect of 500 ml of 7.2 % HS combined with 6 % poly-(O-2-hydroxyl) starch solution over 24 h in 19 adults.^48^ Control groups varied: two studies used either ringer lactate or no specified fluid,^48^, ^49^ whereas the others compared HES to HHS. Further, the time points of fluid administration differed between either, after emergency medical services had established venous access,^46^, ^47^, ^49^ or at arrival to hospital.^48^ ROSC and hospital admission were used either as inclusion criteria^48^ or outcome parameters.^46^, ^47^, ^49^ Survival rate was described either to hospital admission,^46^, ^49^ hospital discharge^47^ or up to one year after resuscitation.^48^ Neurologic outcome was only reported by one study showing a higher proportion of patients with CPC scores of 1–2 in the HHS group.^47^ Infusion volumes and rates were inconsistently reported, and none of the studies incorporated advanced physiologic monitoring. Study details, characteristics and outcomes are summarized in Table 1.

**Table 1:** Human study details and characteristics. Outcomes are in percent of the entire study cohort. HHS: hypertonic saline with hydroxyethyl starch, HES: hydroxyethyl starch, HS: hypertonic saline, CPR: cardiopulmonary resuscitation, ROSC: return of spontaneous circulation, RL: ringer lactate, CPC: cerebral performance category.

**Author, year, first page**	**Country**	**Study type**	**Sample size, observation period**	**Fluid, dose, time point**	**Main Outcome**	**Results**
Bender, 2007, 74	Germany	Randomized, preclinical pilot study	66, hospital admission	HHS (7.2 % NaCl + 6 % HES) or HES, both 2 ml/kg, during CPR	ROSC and hospital admission	ROSC: HHS 66.7 %, HES 51.5 %, *p* = 0.21 hospital admission: HHS 57.6 %, HES 39.4 %, *p* = 0.14

Breil, 2012, 347	Germany	Randomized study	203, hospital discharge	HHS (7.2 % NaCl + 6 % HES) or HES, both 2 ml/kg, during CPR	Survival, hospital admission, neurological	ROSC: HHS 50 %, HES 47.6 % 24-h survival: HHS 42 %, HES 41.7 % hospital discharge survival: HHS 22 %, HES 22.3 % neurological outcome at discharge: CPC1-2: HHS 13 %, HES 4.9 % and CPC 3–4: HHS 9 %, HES 17.5 %, *p* < 0.05, OR 2.929

Hahn, 2014, 628	Germany	Retrospective case-control study	644, hospital admission	HHS (7.2 % NaCl + 6 % HES, 2 ml/kg or none, during CPR	ROSC	ROSC: HHS 59 %, 42.2 % control, *p* < 0.0001

Heradstveit, 2010, 29	Norway	Prospective, randomized controlled study	19, 1 year	HS (7.2 % NaCl + 6 % poly (O-2-hydroxyethyl) starch or RL500 ml/24 h, at hospital admission	Survival	1-year survival: 80 % HS, 78 % control

**Table 2:** Animal study details and characteristics. When available, the osmolarity or molarity of the administered solutions is provided. Values were taken directly from the original manuscrupts or inferred from the described composition. HSL: Hypertonic sodium lactate, HHS: hypertonic saline with hydroxyethyl starch, HES: hydroxyethyl starch, HS: hypertonic saline, CPR: cardiopulmonary resuscitation, ROSC: return of spontaneous circulation, RL: ringer lactate, HSD: 7.5 % saline, 6 % dextran 70, NS: normal saline, cTnI: cardiac Troponin I, BGA: blood gas analysis, HS-5/HS-10: hydrogen-rich saline, CePP: cerebral perfusion pressure, TPA: tissue-type plasminogen activator, CBF: cerebral blood flow, MB: methylene blue, HSL: hypertonic sodium lactate, NDS: Neurological deficit score, TPA: Tissue-type Plasminogen-activator.

**Author, year,** **first page**	**Country**	**Study type**	**Sample size, observation period**	**Fluid, dose, time point**	**Main Outcome**	**Results**
Annoni, 2023, 161	Belgium	Experimental case-control	35, 12 h	NS 20 ml + RL or HSL (0.5 M) 10 mmol + HSL (0.5 M) 30ymol/kg/min or NS 20 ml + HSL (0.5 M) 30ymol/kg/min post-ROSC	Brain, cardiovascular function	Improved haemodynamics in HSL, *p* < 0.001
Bertsch, 2001, 153	Germany	Experimental case-control	10 swine, 3 h	HHS (7.2 % NaCl + 10 % HES) or NS, no specified dose, after ROSC	cTnI	cTnI lower in HHS group, *p* < 0.05
Breil, 2003, 307	Germany	Experimental case-control	34 swine, 4 h	HS (7.2 % NaCl) or HHS (6 % HES + 7.2 % NaCl) or HES (6 % HES) or NS, all 2 ml/kg, onset of CPR	ROSC	Increased resuscitation success and survival with HS and HHS (*p* < 0.05)
Fischer, 1996, 227	Germany	Experimental case-control	14 cats, 30 min	HES (7.5 % NaCl + 6 % HES), 2 ml/kg, or NS, onset of CPR	ROSC	ROSC in every animal. Significant reduction of cerebral no-reflow with HES, *p* < 0.05
Fischer, 2002, 296	Germany	Experimental case-control	21 swine, 2 h	HS (7.2 % NaCl), 2 ml/kg, or HS (7.2 % NaCl), 4 ml/kg, or NS, onset of CPR	ROSC	Increased resuscitation success (*p* < 0.05) and survival (*p* < 0.05) with HS
Gazmuri, 1990, 482	USA	Experimental case-control	25 swine, 24 h	NaHCO_3_ or Na_2_CO_3_ or NS, all 2.5 ml/kg, during CPR	BGA	Increased pH and HCO_3_ with NaHCO_3_ and Na_2_CO_3_
Huo, 2014, 368	China	Experimental case-control	194 rats, 7 d	HS-5 (0.85 mmol/l), 5 ml/kg, or HS-10 (0.85 mmol/l), 10 ml/kg or NS, 10 ml/kg, before CPR	Survival	Improved survival (*p* = 0.021) and neurologic outcome with HS (*p* < 0.0001)
Kaakinen, 2006, 183	Finland	Experimental case-control	24 swine, 7 d	HSD (7.5 % NaCl + 6 % dextran) or NS, both 4 ml/kg, onset of rewarming	Survival	Survival not significant, significant neurologic recovery with HSD (*p* > 0.9)
Kim 2021, 224	Republic of Korea	Experimental case-control	12 swine, 20 min	hypertonic crystalloid or isotonic crystalloid or no fluid, dose/concentration not specified, during CPR	CePP	Higher CePP in hypertonic crystalloid group
Krep, 2003, 337	Germany	Experimental case-control	12 cats, 6 h	HHS (7.5 % NaCl + 6 % HES) + TPA or RL, both 2 ml/kg, during CPR	Cerebral reperfusion	Improved initial cerebral recirculation with HHS + TPA
Krep, 2004, 73	Germany	Experimental case-control	34 swine, 4 h	HS (7.2 % NaCl) or HHS (7.2 % NaCl + 6 % HES) or isooncotic HES (6 %) or NS, 2 ml/kg, onset of CPR	CBF	Improved CBF with HS and HHS, *p* < 0.01
Krieter, 2002, 1031	Germany	Experimental case-control	15 swine, 4 h	HHS (7.2 % NaCl + 10 % HES) or NS, 125 ml, upon ROSC	cTnI and S-100	Reduced increase of S-100 and cTnI with HHS, *p* < 0.05
Liu, 2002, 537	Sweden	Experimental case-control	30 swine, 24 h	HSD (7.5 % NaCl + 6 % dextran)) or NS or sham, 3 ml/kg, during CPR	ROSC, neurologic outcome	More rapid ROSC (*p* = 0.046) and better neurological outcome with HSD (*p* < 0.001)
Miclescu, 2006, 2806	Sweden	Experimental case-control	63 swine, 4 h	MB (7.5 m/kg/h) + HSD 7.2 % NaCl + 6 % dextran (10 m/kg/h) or HSD 7.2 % NaCl + 6 % dextran (10 mL/kg/h) or NS (55 mL/kg/h), during CPR	Survival	increased survival with MB-HSD, *p* = 0.02
Miclescu, 2007, 88	Sweden	Experimental case-control	60 swine, 4 h	NS (55 ml/kg/h) + MB or HSD (7.5 % NaCl + 6 % dextran) 10 ml/kg/h + MB during CPR	Survival	No difference in survival
Miclescu, 2013, 256	Sweden	Experimental case-control	35 swine, 3 h	cold HSD (7.5 % NaCl + 6 % dextran) 3 ml/kg or cold RL 30 ml/kg, upon ROSC	Time to target temperature	Similar time to hypothermia
Nakayama, 2016, 702	USA	Experimental case-control	137 mice, 24 h	3 % HS or 5 % HS or 7.5 % HS or NS, all 1 ml/kg/h, 30 min after ROSC	AQP-4 expression	No difference in AQP-4 expression
Noppens, 2012, 2149	Germany	Experimental case-control	59 rats, 7 d	HS-HES (7.2 % NaCl + 6 % HES) or NS, 4 ml/kg, after ROSC	Local CBF	Improved local CBF with HS-HES, *p* < 0.001
Nozari, 1999, 27	Sweden	Experimental case-control	32 swine, 3.5 h	HSD (7.5 % NaCl + 6 % dextran) + aortic balloon occlusion or HSD (7.5 % NaCl + 6 % dextran) or NS, all 3 ml/kg, during CPR	CePP	higher CBF during CPR with HSD + aortic balloon occlusion, *p* = 0.0139
Sun, 1996, 2035	Japan	Experimental case-control	40 rats, 4 h	HCO_3_^−^ or Na_2_CO_3_ or Sodium bicarbonate, Carbicarb and tromethamine or NS, all 2.5 ml/kg, during CPR	Myocardial function	improved myocardial function with Na_2_CO_3_ and tromethamine
Zhou, 2017, 5783	China	Experimental case-control	60 rats, 3 d	HS (10 % NaCl) or HES (6 % HES), NS or NS + sham-operation, all 2 ml/h, before cardiac arrest	NDS	highest NDS with HS, *p* = 0.004

Two of the RCTs analyzed small to moderate sample sizes (*n* = 19 and *n* = 66),^46^, ^48^ while the other two employed registry-based matched-pair analyses with substantially larger cohorts (*n* = 203 and *n* = 644).^47^, ^49^ All studies focused on adults with out-of-hospital cardiac arrest of presumed cardiac origin. Although inclusion criteria were broadly comparable, important differences emerged in baseline demographics, exclusion criteria and post-resuscitation care protocols such as hypothermia and coronary intervention strategies.

#### Study outcomes

Overall, short-term outcomes tended to favor hypertonic fluid therapy. The two large registry-based studies reported higher rates of ROSC and hospital admission in patients treated with HHS compared to matched controls.^47^, ^49^ For example, one study found ROSC rates of 59.0 % versus 42.2 % and hospital admission rates of 52.5 % versus 33.5 %.^49^ Only one study observed a statistically significant improvement in favorable neurologic outcome at hospital discharge (13 % vs. 5 %, *p* < 0.05).^47^ By contrast, the post-ROSC RCT found no significant differences in cerebral oedema formation, systemic vascular resistance or neurological imaging findings.^48^

#### Safety of fluids

Despite their differences, all studies in humans demonstrated that HTF/HOF administration was feasible and did not result in overt harm.^46^, ^47^, ^48^, ^49^ Across studies, electrolyte shifts were observed, most prominently transient rises in plasma sodium immediately after infusion.^46^, ^47^ Importantly, these changes tended to normalize within hours and were not associated with clinically relevant arrhythmias, neurologic deterioration or renal failure.^48^, ^49^ Similarly, haemodynamic tolerance was good: blood pressure and heart rate remained stable and vasopressor requirements did not increase compared to controls.

#### Risk of bias assessment of human studies

Assessment of randomized human trials by ROB-2revealed overall low risks of bias in all categories. The retrospective matched-pair analysis showed some concerns for selection bias and reporting methods using ROBINS-I.

## Discussion

This scoping review summarizes the available evidence on the effects of HTF/HOF administered during or immediately after CPR in both animals and human studies. Overall, results are supportive for using HTF/HOF. Animal experiments consistently demonstrated favorable haemodynamic and neuroprotective effects. Across human studies, hypertonic and hyperoncotic fluids showed variable effects: some trials reported modest improvements in ROSC^47^, ^49^ or early hemodynamic stabilization,^46^, ^48^ while survival to discharge and neurological recovery remained largely unchanged. Reported benefits were generally not statistically significant and should be interpreted as exploratory or hypothesis-generating rather than clinically conclusive. Given the limited number of clinical trials and the considerable heterogeneity across study designs and outcomes, the evidence remains insufficient to draw definitive conclusions. In the following, we discuss the potential implications for clinical practice and priorities for future research.

### Animal studies

Despite substantial heterogeneity of studies, animal models have provided fundamental insights into the physiological effects and potential therapeutic benefits of HTF/HOF in cardiac arrest. Across species and protocols, small-volume HTF/HOF showed consistent haemodynamic and neuroprotective advantages including improved myocardial and cerebral blood flow, reduced myocardial and cerebral injury,^36^, ^49^ reduced cerebral no-reflow, increased rates of ROSC and short-term survival.^26^, ^35^, ^42^ These effects of small-volume fluids are likely linked to improved microcirculation, reduced endothelial swelling and partial buffering of metabolic acidosis. By contrast, large volumes of isotonic fluids lowered perfusion pressures and impaired resuscitation outcomes.^27^ Importantly, doubling the hypertonic dose did not provide further benefit, suggesting that the effect relies more on osmotic and microcirculatory mechanisms than on intravascular volume expansion.^42^ In line with this, ERC guidelines explicitly advise against liberal fluid loading during cardiac arrest.^3^

#### Implications for translational research

The findings from animal studies provide a compelling experimental basis for using HTF/HOF in cardiac arrest, closely aligned with the pathophysiological challenges of resuscitation. Future studies should aim to optimize the composition of hypertonic and hyperoncotic fluids, determine the optimal timing of administration and identify patient subgroups that may benefit most. Furthermore, ROSC is a weak endpoint in experimental cardiac arrest models, as many protocols are designed to achieve ROSC. Variability in model characteristics, rather than treatment effects may therefore account for observed differences in ROSC rates across studies. Future animal studies should emphasize translational endpoints, such as neurologic recovery, delayed inflammation, and blood–brain barrier integrity. Biomarkers such as neuron-specific enolase or S100B and neuroimaging modalities may provide more reproducible indicators of cerebral injury and recovery. Harmonization of protocoels and use of standardized core outcomes could facilitate synthesis across studies and accelerate the bench-to-bedside translation.

### Human studies

The four human studies collectively support the feasibility and safety of application of HTF/HOF in cardiac arrest. The available evidence suggests that short-lived electrolyte disturbances and the haemodynamic profile of hypertonic solutions are manageable. Beyond this, two important areas remain for discussion, the timing of administration and the composition of fluids.

#### Timing of fluid therapy

The clinical trials administering HHS intra-arrest, mostly immediately after vasopressor use, aimed at improving myocardial and cerebral perfusion during the low-flow state auf CPR.^46^, ^47^, ^49^ This approach targets circulatory compromise as a determinant of resuscitation success and was associated with higher rates of ROSC and hospital admission.^49^ By contrast, the trial starting HHS only after ROSC^48^ was based on the rationale of reducing post-ischemic capillary leakage. To clarify and differentiate mechanisms of action, future studies should test the hypothesis that intra-arrest administration of HTF/HOF may better preserve cerebral perfusion and neurological outcomes compared with delayed post-ROSC infusion, due to its earlier impact on microcirculatory flow and reperfusion quality.

#### Composition of fluids

Most trials investigated HHS, i.e. 7.2 % HS combined with 6 % HES, whereas one RCT evaluated HS alone.^47^ The addition of starch intended to prolong intravascular retention and counteract leakage, potentially enhancing the haemodynamic effect. While the clinical studies did not consistently demonstrate survival benefits, registry data suggest that combined hypertonic/hyperoncotic therapy may improve short-term outcomes.^49^ However, starch-based solutions such as HES have been associated with adverse effects such as kidney injury and coagulation disturbances, which may limit their clinical applicability. This prompted regulatory restrictions by the European Medicines Agency (EMA), but whether these safety concerns apply to short-term, intra-arrest administration of small amounts of fluids remains unclear and warrants specific evaluation.^50^ In contrast, HS alone may offer osmotic benefits without the colloid’s properties of stabilizing capillary leak or introducing potential adverse effects. Taken together, the influence of fluid compositions on different patient groups and time points of administration needs further investigation. Further, the hemodynamic and osmotic effect of HTF/HOF scale with total solute load rather than infusion volume alone. Reporting administered dose and osmolarity would allow better comparison between hypertonic saline, dextran, starch and lactate-based solutions. Beyond osmotic and oncotic properties, the ionic and metabolic composition of hypertonic fluids may critically influence their physiological effects during and after cardiac arrest. The chloride load associated with hypertonic saline may exacerbate metabolic acidosis in a setting where base deficit is already pronounced. Conversely, bicarbonate-based hypertonic formulations, although intended to buffer acidosis, may paradoxically worsen intracellular acidosis due to CO_2_ generation and impaired clearance during hypoxia. In contrast, metabolically active or pH-neutral solutions such as hypertonic lactate, ketone bodies, or starch-based fluids may differ in their effects on acid–base homeostasis and post-resuscitation recovery. Future studies comparing such types of solutions under standardized conditions are needed to delineate their respective benefits and risks.

### Overall evidence and clinical implications

The combined evidence from human and animal studies presents a cautiously optimistic view of HTF/HOF therapy in cardiac arrest. Preclinical animal studies consistently demonstrate physiological and neuroprotective benefits while translation to cardiac arrest in humans is limited. This translational gap appears vital to be closed by more rigorous human trials that include downstream outcomes such as biomarkers and imaging.

#### Evidence in current guidelines

Treatment recommendations by the International Liaison Committee on Resuscitation (ILCOR) and the European Resuscitation Council (ERC) Guidelines, do *not* endorse HTF/HOF as a routine intervention in cardiac arrest management.^3^, ^51^ ILCOR highlights that the available evidence is insufficient to support or refute the routine use of fluid infusion during cardiac arrest, emphasizing the lack of consistent benefit across clinical studies. Similarly, the ERC guidelines note that liberal fluid administration may be harmful and recommend a restrictive approach, without endorsing hypertonic solutions for routine intra-arrest or post-ROSC therapy. This underscores that HTF/HOF should remain investigational in cardiac arrest, and routine use outside of research settings is premature.

#### Future research directions

Based on the available evidence, future trials should employ multicentre RCT designs with adequate power, harmonized outcome definitions, and robust neurological follow-up. The use of biomarkers, cerebral perfusion imaging, and stratification by arrest type (e.g., shockable vs. non-shockable rhythms) may enhance our pathophysiological understanding and support tailored patient selection. Ultimately, clarifying whether early, targeted fluid therapy can improve neurological outcomes will be central to defining the role of HTF/HOF in cardiac arrest management.

### Limitations

First, despite thorough evaluation of our inclusion and exclusion criteria, eligible studies might have been lost. As we excluded papers and abstracts published in other languages than English, we could possibly have missed similar data. No subgroup analysis could be performed based on cardiac arrest etiology and pediatric data were not included despite potential relevance. Moreover, trauma-related cardiac arrest was excluded to maintain conceptual focus, which limits generalizability to that population. Further, the low number and the considerable heterogeneity of available studies precluded performing a systematic review or specific meta-analyses. Moreover, no new human studies on this topic have been published in the past years, further underscoring the existing evidence gap. Finally, both animal and human studies were conceptually grounded in variable resuscitation practices, including differences in CPR technique, adjunctive medications and post-ROSC care. As a result, the generalizability of these findings to current clinical practice is limited.

## Conclusion

Findings suggest potential benefits for ROSC and early neurological outcomes, particularly when administered intra-arrest in animal models. Human data are emerging but remain inconclusive, underscoring the need for well-designed clinical trials to confirm translational relevance. However, substantial heterogeneity in study designs, timing, and outcome measures limits definitive conclusions. Rigorous, adequately powered clinical trials with standardized endpoints are therefore warranted to clarify the therapeutic potential and clinical role of HTF/HOF in cardiac arrest management.

## Declaration of generative AI and AI-assisted technologies in the writing process

During the preparation of this work the authors used ChatGPT in order to check the paper for conciseness. After using this tool, the authors reviewed and edited the content as needed and take full responsibility for the content of the published article.

## CRediT authorship contribution statement

**Samira Akbas:** Writing – review & editing, Writing – original draft, Visualization, Methodology, Investigation, Formal analysis, Data curation. **Clara Castellucci:** Writing – review & editing, Writing – original draft, Visualization, Methodology, Investigation, Formal analysis, Data curation, Conceptualization. **Christopher Haresleb:** Writing – review & editing, Visualization, Investigation, Formal analysis. **Alexander Kaserer:** Writing – review & editing, Investigation, Formal analysis, Data curation. **Donat R. Spahn:** Writing – review & editing, Supervision. **Jan Breckwoldt:** Writing – review & editing, Supervision, Methodology, Investigation, Data curation, Conceptualization.

## Funding

This academic study received no funding.

## Declaration of competing interest

**Alexander Kaserer** received lecture honoraria from Bayer AG (Switzerland), CSL Behring GmbH (Switzerland) and advisory honoraria from AstraZeneca AG (Switzerland) and Pharmacosmos (Switzerland).

**Donat R. Spahn** is chair of the ABC-Trauma Faculty, sponsored by unrestricted educational grants from Alexion Pharma Germany GmbH, Munich, Germany, CSL Behring GmbH, Marburg, Germany, and LFB Biomédicaments, Courtaboeuf Cedex, France. Dr. Spahn is also the president of Alliance Rouge, Bern, Switzerland, CEO of Swiss-PBM-Consulting GmbH, Zurich, Switzerland and a member of the Advisory Board of Saipient AG, Zurich, Switzerland.

Dr. Spahn received honoraria / travel support for consulting or lecturing from:

Alliance Rouge, Bern, Switzerland, Danube University of Krems, Austria, European Society of Anesthesiology and Intensive Care, Brussels, BE, International Foundation for Patient Blood Management, Basel, Switzerland, Korean Society of Anesthesiologists, Seoul, Korea, Network for the Advancement of Patient Blood Management, Haemostasis and Thrombosis, Paris, France, Society for the Advancement of Blood Management, Mount Royal NJ, Alexion Pharmaceuticals Inc., Boston, MA, AstraZeneca AG, Baar, Switzerland, Baxter AG, Glattpark, Switzerland, Bayer AG, Zürich, Switzerland, B. Braun Melsungen AG, Melsungen, Germany, CSL Behring GmbH, Hattersheim am Main, Germany and Berne, Switzerland, CSL Vifor (Switzerland) Villars-sur-Glâne, Switzerland, CSL Vifor (International), St. Gallen, Switzerland, Celgene International II Sàrl, Couvet, Switzerland, Daiichi Sankyo AG, Thalwil, Switzerland, Haemonetics, Braintree, MA, USA, iSEP, Nantes, France, Novo Nordisk Health Care AG, Zurich, Switzerland, Octapharma AG, Lachen, Switzerland, Pharmacosmos A/S, Holbaek, Denmark, Pierre Fabre Pharma, Alschwil, Switzerland, Portola Schweiz GmbH, Aarau, Switzerland, Roche Diagnostics International Ltd, Reinach, Switzerland, Shire Switzerland GmbH, Zug, Switzerland, Werfen, Bedford, MA, Zuellig Pharma Holdings, Singapore, Singapore.

**Jan Breckwoldt** is member of the ILCOR Task Force ‘Education, implementation and Teams’, member of the Writing group of the ERC Guideline 2025, Chapter ‘Education for Resuscitation’, member of the ERC Committee SEC-EIS. He is also member of the EPA Committee of the Swiss Institute for Medical Education (SIME). and Co-Chair of the ‘Teach the teachers’ program of the SIME.

All other authors have no competing interests to declare.
