# Supplementary material for: Effects of peri-arrest hypertonic/hyperoncotic fluid treatment on clinical outcomes in non-trauma-related resuscitation – A scoping review
Source: Resusc Plus. 2025 Nov 20;27:101171. doi: 10.1016/j.resplu.2025.101171 (PMC12704065; doi:10.1016/j.resplu.2025.101171)
Supplement: Supplementary Data 1 [file mmc1.docx]

**Supplementary Tables**

| ROB 2 | **Author, year,**  **first page** | **Randomisation** | **Deviation from intended interventions** | **Missing outcome data** | **Measurement of the outcome** | **Selection of the reported results** | **Overall** |  |  |
| --- | --- | --- | --- | --- | --- | --- | --- | --- | --- |
|  | Bender, 2007, 74 | Low | Low | Low | Low | Low | Low |  |  |
|  | Breil, 2012, 347 | Low | Low | Low | Concerns (low for survival, high for neurological outcome) | Low | Low |  |  |
|  | Heradstveit, 2010, 29 | Low | Low | Low | Low | Low | Low |  |  |
|  |  |  |  |  |  |  |  |  |  |
| ROBINS I | **Author, year,**  **first page** | **Confounding** | **Selection** | **Classification of intervention** | **Deviation from intended intervention** | **Missing data** | **Measurement**  **of outcomes** | **Selection of reported results** | **Overall** |
|  | Hahn, 2014, 628 | Low | Concerns (possible due to 'local protocols') | Low | Low | Low | Concerns (dependent on local setting) | Low | some concerns |

**Supplementary Table 1:** Grading strategies using ROB2 (Risk of bias assessment 2) and ROBINS-I (Risk of Bias in Non-randomised Studies - of Interventions). Scoring is either low, concerns or high. For concerns, we stated our reasons in brackets.

| **Author, year** | **Random group allocation** | **Baseline group characteristics** | **Allocation concealed** | **Random housing** | **Blinding examiners** | **Random outcome selection** | **Blinding of assessor** | **Attrition (drop-outs)** | **Any randomization** | **Any blinding** | **Sample size calculation** | **Temperature control** |
| --- | --- | --- | --- | --- | --- | --- | --- | --- | --- | --- | --- | --- |
| Breil, M. 2003 | Low | Low | Low | Low | Low | Low | Low | Low | Low | Low | High (not described) | Low |
| Fischer, M. 2002 | Low | Low | Low | Low | Low | Low | Low | Low | Low | Low | High (not described) | Low |
| Kim, KH. 2021 | Low | High (differences in hyper.crist. group) | Low | Low | High (not described) | Low | High (not described) | Low | Low | High (not blinded) | Low | High (not described) |
| Kaakinen, T. 2006 | Low | Low | Low | Low | Low | Low | Low | Low | Low | Low | High (not described) | Low |
| Miclescu, A. 2006 | Low | Low | Low | Low | High (not blinded) | Low | High (not blinded) | Low | Low | High (not blinded) | High (not described) |  |
| Miclescu, A. 2007 | Low | Low | Low | Low | Concerns (some blinded) | Low | Low | Low | Low | Low | Low | Low |
| Bertsch, T. 2001 | Low | Low | Low | Low | Concerns (some blinded) | Low | Low | Low | high | Concerns | High (weight difference of animals) | Low |
| Gazmuri, RJ. 1990 | High (randomization not specified) | High (mixed sex and age) | Low | Low | Low | Low | Low | High (no signig. data on dead animals) | Low | Low | High (weight and age difference of animals) | Low |
| Krieter, H. 2002 | Low | Concerns (differences in characteristics) | Low | Low | Concerns (some blinded) | Low | Low | Low | Low | Low | Concerns (different gender) | Low |
| Krep, H. 2004 | Low | Low | Low | Low | High (not blinded) | Concerns (no data on two animals reported) | Low | Concerns (some data not available) | Low | Low | High (weight difference of animals) | Low |
| Liu, X. 2002 (Wiklund) | Low | Low | Low | Low | Concerns (balloon occlusion) | n.a. | Low | Low | Low | High (not blinded for balloon occlusion) | Low | Low |
| Nozari, A. 1999 (Wiklund) | Low | Low | Low | Low | Concerns (balloon occlusion) | n.a. | Low | Low | Low | High (not blinded for balloon occlusion) | Low | Low |
| Miclescu, A. 2013 | Low | Low | Low | Low | Concerns (two groups with hypothermia) | n.a. | Low | Low | Low | hypothermia (cool packs) | Low | Low |
| Sun, S. 1996 | Low | Low | Low | Low | Concerns (4min vs. 8min of CA) | n.a. | High (not described) | Low | Low | Concerns (unclear if solutions blinded and durations not blinded) | Low | n.a. |
| Zhou, X. 2017 | Low | Low | Low | Low | High (not described) | n.a. | Low | Low | Low | Concerns (unclear if solutions blinded) | Low | Low |
| Huo, TT. 2014 | Low | Low | Low | Low | High (not described) | Low | Low | Low | Low | Low | High (not described) | Low |
| Noppens, R. 2012 | Low | Low | Low | Low | High (not described) | Low | Low | Low | Low | Low | High (not described) | Low |
| Fischer, M. 1996 | Low | High (CPP, MPP differs significantly) | Low | Low | High (not described) | Low | High (not described) | Low | Low | High (CPR by one investigator) | High (not described) | Low |
| Krep, H. 2003 | Low | High (Baseline Glucose differs significantly) | Low | Low | High (not described) | Low | High (not described) | Low | Low | Low | High (not described) | Low |
| Nakayama, S. 2016 | Low | Low | Low | Low | High (not described) | Low | Low | Low | Low | Low | High (not described) | Low |
| Annoni, F. 2023 | Low | Low | Concerns (no information on allocation-blinding) | Low | High (not blinded) | Low | Low | Low | Low | Concerns (not all measures blinded) | Hgih (only convenient sample size) | Low |

**Supplementary Table 2: Risk of Bias Assessment of Animal Studies Using the SYRCLE Tool**

Risk of bias assessment of all included animal studies was performed using the SYRCLE risk of bias tool (25), which assesses internal validity across domains of selection, performance, detection, attrition, reporting, and other sources of bias. Ratings are expressed as 'Low', 'High', or 'Unclear' (if information was insufficient).

| **Section** | **Item** | **Description in manuscript** | **Location** |
| --- | --- | --- | --- |
| TITLE | 1. Title | Identifies the report as a scoping review. | Title page |
| ABSTRACT | 2. Abstract | Structured abstract with background, objectives, methods, results, and conclusions. | Abstract |
| INTRODUCTION | 3. Rationale | Provides background and rationale for evaluating HTF/HOF use in cardiac arrest. | Introduction |
| INTRODUCTION | 4. Objectives | States aim to map existing evidence on HTF/HOF during CPR and identify knowledge gaps. | Introduction – final paragraph |
| METHODS | 5. Protocol and registration | Conducted according to PRISMA-ScR framework; no prior registration. | Methods – Study design |
| METHODS | 6. Eligibility criteria | Describes inclusion and exclusion criteria for human and animal studies. | Methods – Eligibility criteria |
| METHODS | 7. Information sources | Lists databases (PubMed, Embase, Web of Science) and time frame searched. | Methods – Search strategy |
| METHODS | 8. Search | Provides detailed search strategy in supplementary material. | Supplementary File 1 |
| METHODS | 9. Selection of sources | Describes study selection process; depicted in PRISMA flow diagram. | Methods – Study selection |
| METHODS | 10. Data charting process | Explains data extraction by two independent reviewers. | Methods – Data extraction and synthesis |
| METHODS | 11. Data items | Lists variables extracted: species, intervention, comparator, outcomes, and bias assessment. | Methods – Data extraction and synthesis |
| METHODS | 12. Critical appraisal | Details bias assessment using ROB2, ROBINS-I, and SYRCLE tools. | Methods – Risk of bias assessment |
| METHODS | 13. Synthesis of results | States results were synthesized narratively with descriptive statistics; no meta-analysis performed. | Methods – Data synthesis |
| RESULTS | 14. Selection of sources | Presents number of records screened, excluded, and included (PRISMA diagram). | Results – Study selection; Figure 1 |
| RESULTS | 15. Characteristics of sources | Summarizes study characteristics in Tables 1 (human) and 2 (animal). | Results – Overview of included studies |
| RESULTS | 16. Critical appraisal | Summarizes bias assessment findings; Supplementary Table 2 lists SYRCLE results. | Results – Risk of bias section |
| RESULTS | 17. Results of individual sources | Describes findings from individual studies grouped by population and intervention. | Results – Human and animal studies |
| RESULTS | 18. Synthesis of results | Provides narrative synthesis by outcome type and timing (intra-arrest vs. post-ROSC). | Results – Summary section |
| DISCUSSION | 19. Summary of evidence | Summarizes key findings, translational implications, and gaps. | Discussion – Overview |
| DISCUSSION | 20. Limitations | Discusses limitations: heterogeneity, language bias, lack of subgroup analyses, exclusion of trauma. | Discussion – Limitations section |
| DISCUSSION | 21. Conclusions | Highlights translational potential and need for targeted clinical trials. | Discussion – Conclusion paragraph |
| FUNDING | 22. Funding | States no external funding or industry support. | End of manuscript |

**Supplementary Table 3: PRISMA-ScR-Checklist**

This checklist summarizes how the PRISMA-ScR (Preferred Reporting Items for Systematic Reviews and Meta-Analyses extension for Scoping Reviews) criteria were addressed in the manuscript.
